# Supplementary material for: A sliding window approach to optimize the time-varying parameters of a spatially-explicit and stochastic model of COVID-19
Source: PLOS Glob Public Health. 2022 Sep 15;2(9):e0001058. doi: 10.1371/journal.pgph.0001058 (PMC10021528; doi:10.1371/journal.pgph.0001058)
Supplement: S1 Text — (DOCX) [file pgph.0001058.s001.docx]

# S1 Text

A Sliding Window Approach to Optimize the Time-varying Parameters of a Spatially-explicit and Stochastic Model of COVID-19

Saikanth Ratnavale *et al.*

The following set of ordinary differential equations describes the dynamics within each sub-population of the model:

$$\begin{matrix} \frac{dS}{dt} & =-\beta_{t}\lambda_{t}S-m_{t}S \\ \frac{dE}{dt} & =\beta_{t}\lambda_{t}S-\delta_{1}E \\ \frac{dI_{a}}{dt} & =\delta_{1}\rho_{1}E-\gamma_{a}I_{a}-m_{t}I_{a} \\ \frac{dI_{p}}{dt} & =\delta_{1}\left( 1-\rho_{1} \right)E-\delta_{2}I_{p}-m_{t}I_{p} \\ \frac{dI_{s}}{dt} & =\delta_{2}I_{p}-\delta_{3}I_{s}-m_{t}I_{s} \\ \frac{dI_{b}}{dt} & =\delta_{3}\left( 1-\rho_{2}-\rho_{3} \right)I_{s}-\gamma_{b}I_{b} \\ \frac{dI_{h}}{dt} & =\delta_{3}\rho_{2}I_{s}-\delta_{4}I_{h} \\ \frac{dI_{c1}}{dt} & =\delta_{3}\rho_{3}I_{s}+\delta_{4}\rho_{4}I_{h}-\delta_{5}I_{c1} \\ \frac{dI_{c2}}{dt} & =\delta_{5}\left( 1-\rho_{5} \right)I_{c1}-\gamma_{c}I_{c2} \\ \frac{dD}{dt} & =\delta_{5}\rho_{5}I_{c1} \\ \frac{dR}{dt} & =\gamma_{a}I_{a}+\gamma_{b}I_{b}+\gamma_{c}I_{c2}+\delta_{4}\left( 1-\rho_{4} \right)I_{h} \end{matrix}$$

and $\lambda_{t}$ represents a component of the force of infection, given by

$$\lambda_{t}=\frac{\omega_{1}I_{a}+I_{p}+I_{s}+I_{b}+\omega_{2}\left( I_{h}+I_{c1}+I_{c2} \right)}{N-D}.$$

**Table A:** COVID-19 model state variables

| **State Variable** | **Description** |
| --- | --- |
| $S$ | Number of susceptible individuals |
| $E$ | Number of exposed individuals |
| $I_{a}$ | Number of asymptomatic individuals |
| $I_{p}$ | Number of pre-symptomatic individuals |
| $I_{s}$ | Number of mildly symptomatic individuals |
| $I_{b}$ | Number of mildly symptomatic individuals on bed rest at home |
| $I_{h}$ | Number of hospitalized individuals |
| $I_{c1}$ | Number of individuals in the ICU |
| $I_{c2}$ | Number of individuals in the recovery (step-down) ICU |
| $D$ | Number of deceased individuals |
| $R$ | Number of recovered individuals |
| $N$ | Total number of individuals in the population |

**Table B:** COVID-19 model parameter descriptions

| **Parameter** | **Description** | **Value** |
| --- | --- | --- |
| $\beta_{t}$ | Time-varying transmission rate | Variable |
| $m_{t}$ | Time-varying movement rate | Variable |
| $\omega_{1}$ | Proportion reduction in transmission for asymptomatic folks | 0.55 |
| $\omega_{2}$ | Proportion reduction in transmission for hospitalized folks | 0.05 |
| $\delta_{1}$ | Transition rate: exposed to pre-symptomatic | 1/3.0 |
| $\delta_{2}$ | Transition rate: pre-symptomatic to symptomatic | 1/2.0 |
| $\delta_{3}$ | Transition rate: symptomatic to home or regular hospital bed or ICU | 1/6.0 |
| $\delta_{4}$ | Transition rate: regular hospital bed to home or ICU | 1/7.0 |
| $\delta_{5}$ | Transition rate: ICU to step-down ICU or decease | 1/8.0 |
| $\gamma_{a}$ | Recovery rate: asymptomatic | 1/6.0 |
| $\gamma_{b}$ | Recovery rate: home bed | 1/3.0 |
| $\gamma_{c}$ | Recovery rate: step-down ICU | 1/4.0 |
| $\rho_{1}$ | Fraction of exposed that transition to asymptomatic | 0.28 |
| $\rho_{2}$ | Fraction of symptomatic that transition to hospital bed | 0.20 |
| $\rho_{3}$ | Fraction of symptomatic that transition directly to ICU bed | 0.015 |
| $\rho_{4}$ | Fraction of hospitalized that transition to ICU | 0.28 |
| $\rho_{5}$ | Fraction of patients in ICU that die of disease | 0.60 |
| $\phi$ | Constant controlling the dispersal kernel shape | 25.0 |
| $\sigma$ | Stochasticity standard deviation for transmission rate (stoch_sd) | 0.05 |

**Table C:** Algorithm for grid search with sliding window technique

| **Algorithm** |
| --- |
| 1. Read raw data, decide number of days to consider. 2. Set parameter min/max, window_size and compute n_windows. 3. Set win_count = 0, param_temp and n_searches. 4. Compute temporary maximum likelihood: temp_max, initialize best parameter set. 5. Set maximum likelihood: max_lhood = temp_max. 6. Set search_ID = 0. 7. Compute number of steps and step size. 8. Set index = starter. 9. Set param = min. 10. Compute temp_max. 11. Update best parameter set for search_ID if temp_max >= max_lhood. 12. Stop if param = max, or set param = param + step size and return to Step 10. 13. Reset param_temp to current best parameter set. 14. Stop, or set index = index + 1 and return to Step 9. 15. Stop if search_ID = n_searches, or set search_ID = search_ID + 1 and return to Step 7. 16. Save the best parameter set for the current window. 17. Stop if win_count = n_windows, or set win_count = win_count + 1 and return to Step 4. 18. Calculate the full likelihood for all windows considered. |

Above, we denote n_windows the number of windows for which we calculate the parameter sets. This can be obtained by, first calculating the number of weeks (n_weeks) from the raw data and then by n_windows = n_weeks - window_size + 1. Suppose that, we have $n$ number of weeks with sliding window size of 3 weeks, we find $n-2$ windows or weekly parameter sets (see Figure [3](#fig:sliding-window)). Any remaining days beyond the final window will be included to that window, so the final window size may vary depending on the total number of days. By n_searches, we denote the number of grid searches carried out for one window. For each window, we randomly choose the starting parameter with index (starter) and optimize all the parameters, one at a time.**Supporting Figures**


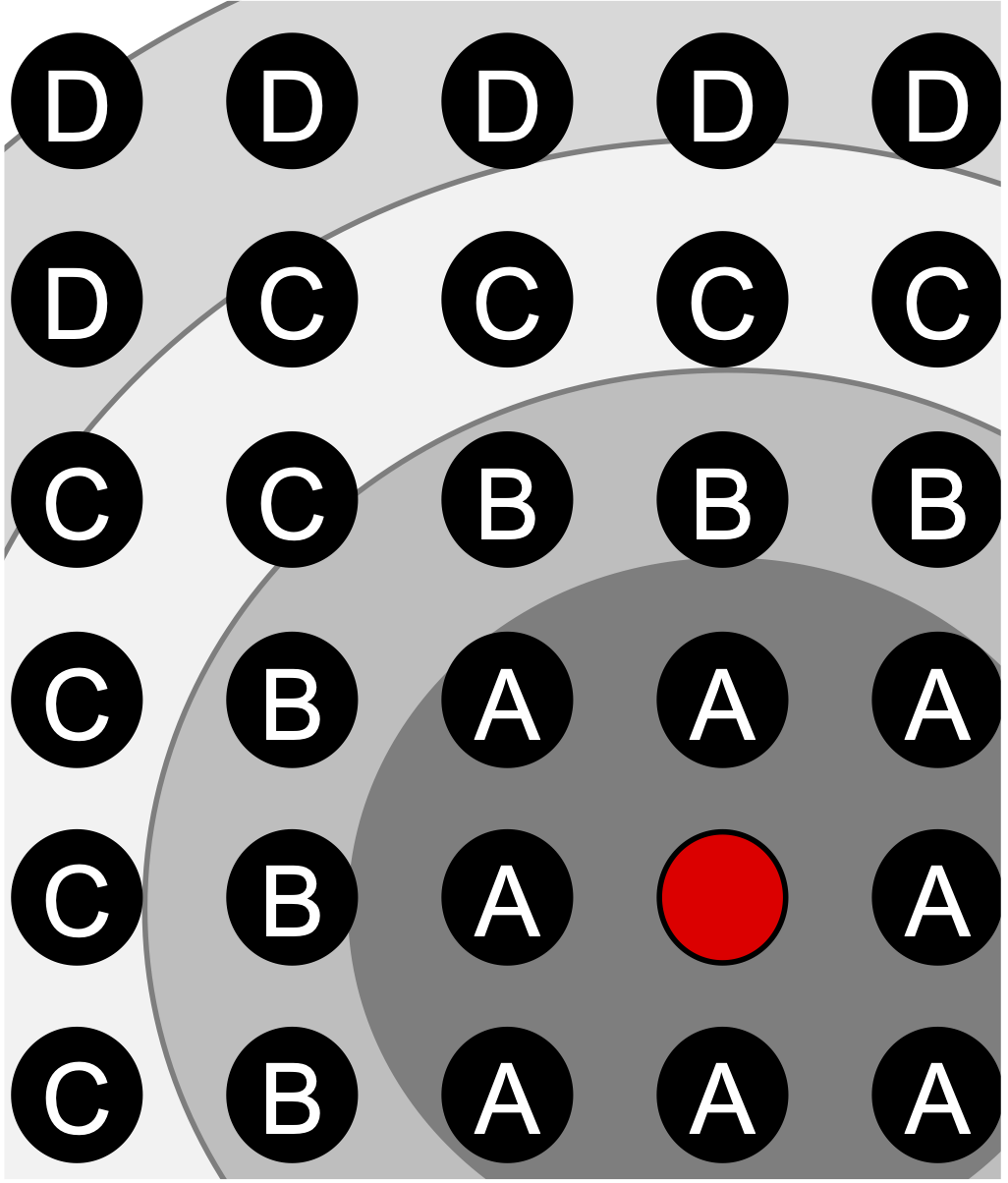


**Fig A:** Schematic that shows how populations were grouped by a 50km buffer around the epicenter sub-population (shown in red), used to calculate the group-based likelihood (equation 4). In this case, hospitalization data from sub-populations in the same group (shown as letters) are added together.


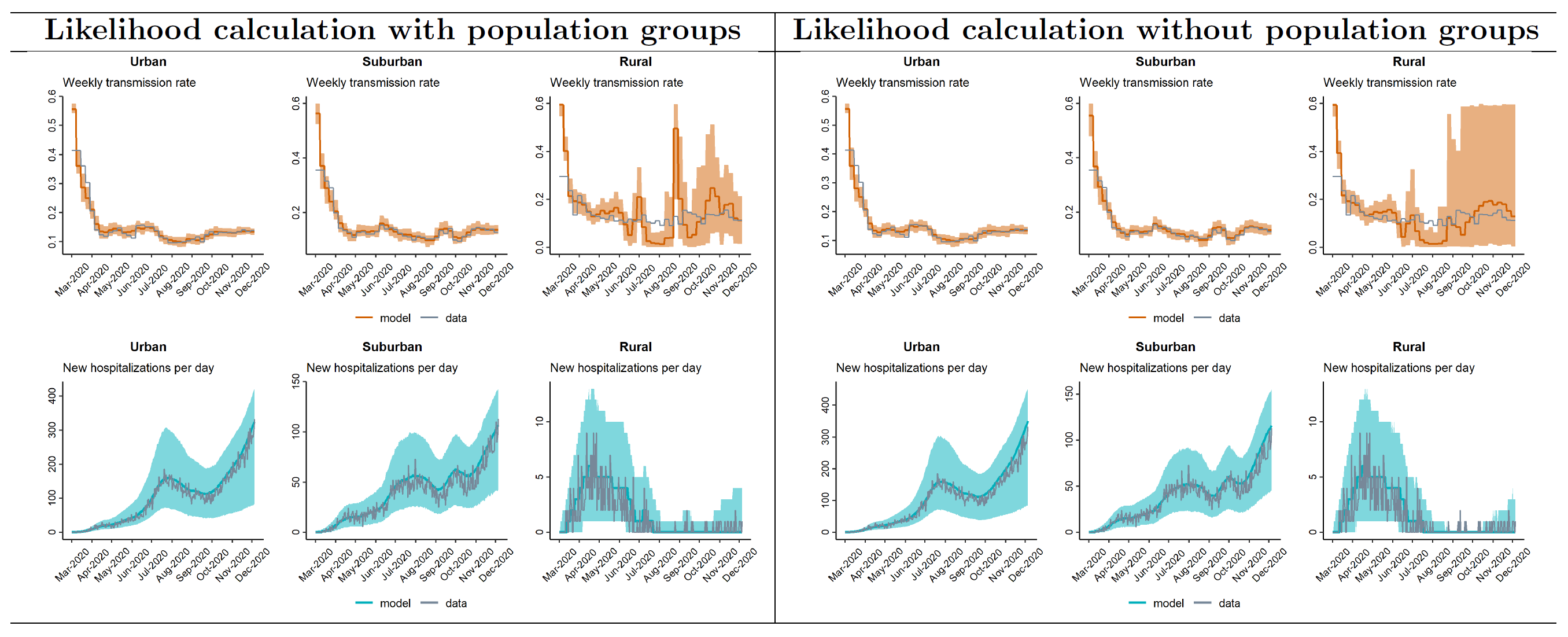


**Fig B:** Estimate of $\beta_{t}$ when $m_{t}$ is known, comparing our two likelihood methods. Note that, although subtle, more error in the estimate of $\beta_{t}$ can be detected when populations are not grouped, particularly in the rural scenario.


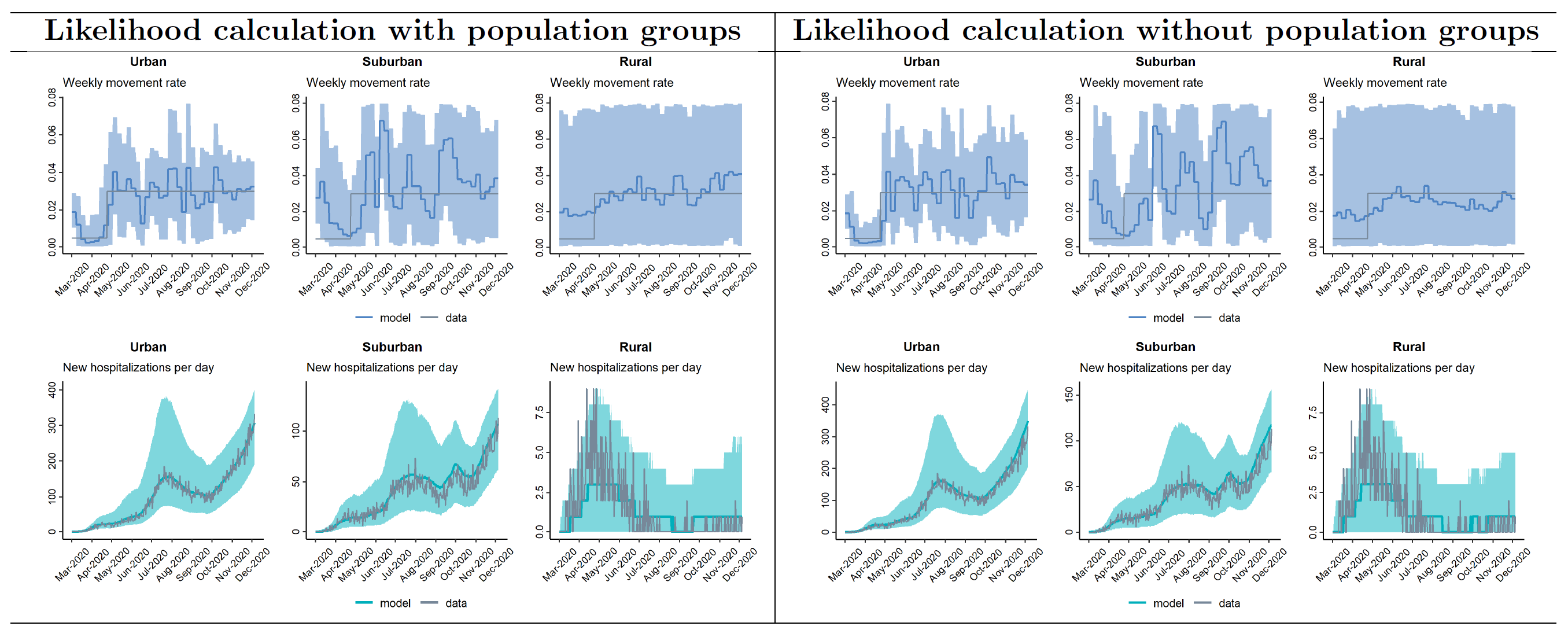


**Fig C:** Estimate $m_{t}$ when $\beta_{t}$ is known, comparing the two likelihood methods. Note that effects of the likelihood method are not strong in this case.


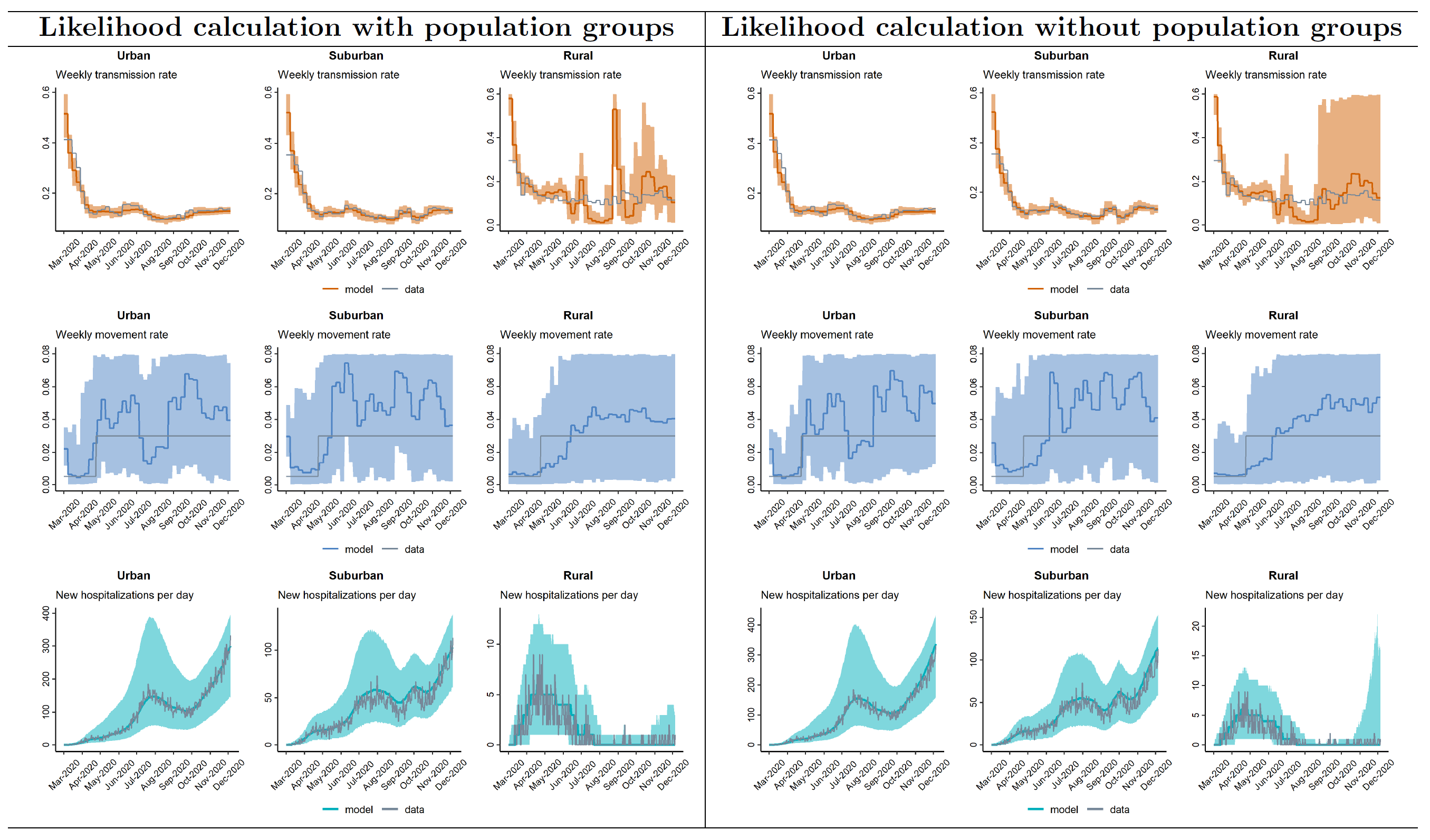


**Fig D:** Estimates of both $\beta_{t}$ and $m_{t}$, comparing the two likelihood methods. By grouping the populations, there is less variation in the parameter estimates, which leads to less variation in how the model fits to the data.
